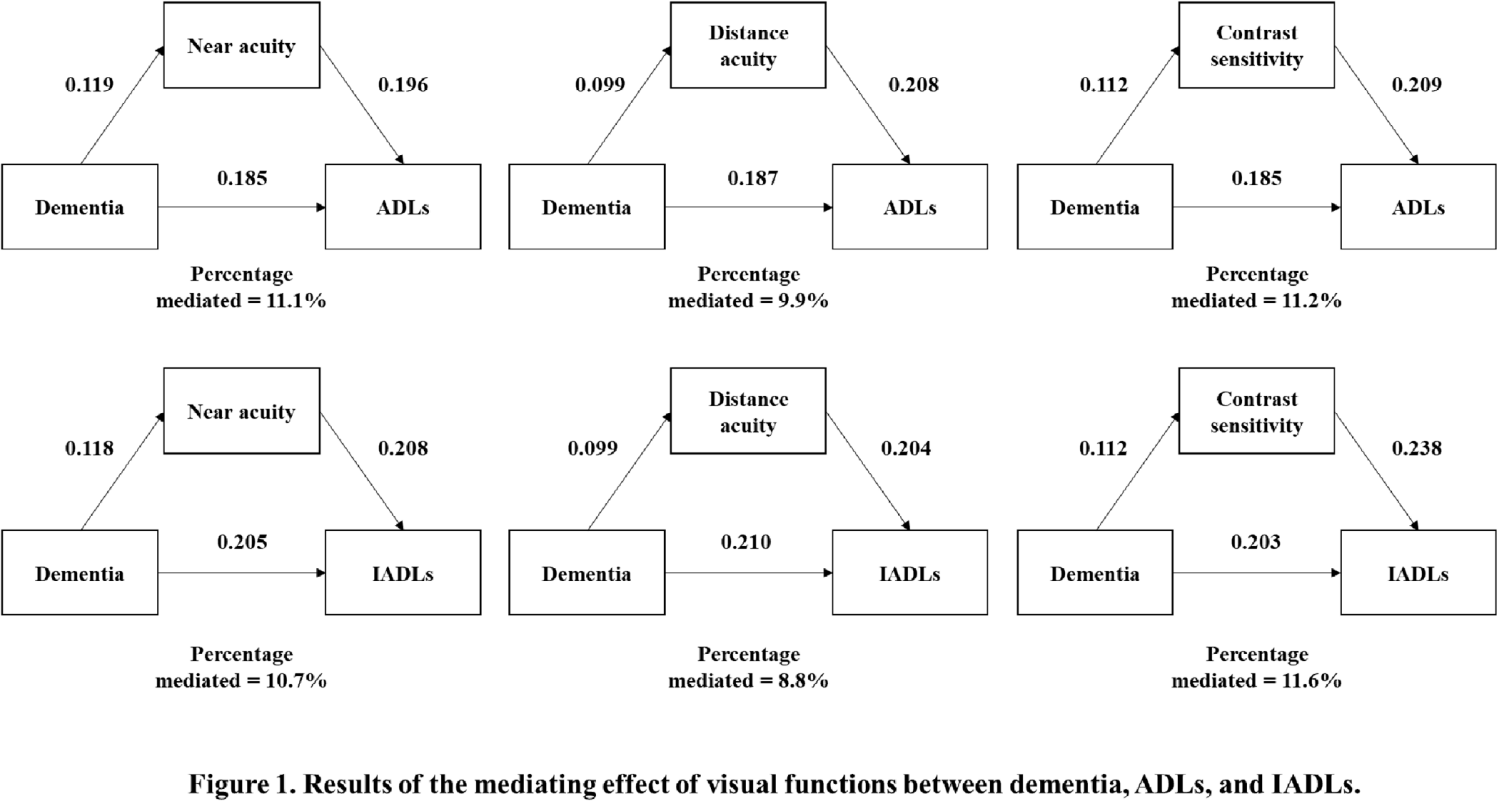# Association between dementia and functional activities of daily living: Mediating effect of visual functions

**DOI:** 10.1002/alz.088616

**Published:** 2025-01-09

**Authors:** Yeonju Jin, Ickpyo Hong

**Affiliations:** ^1^ Yonsei University, Wonju Korea, Republic of (South); ^2^ Yonsei University, Wonju, Gangwon‐do Korea, Republic of (South)

## Abstract

**Background:**

Dementia and visual impairment are both associated with reduced mobility and impaired functioning in activities of daily living (ADL) and instrumental activities of daily living (IADL). Cognitive deficits in older adults have more difficulties in performing daily tasks, increase the risk of fear of participation and may lead to injury (e.g. falls, fractures). However, it is not clear whether visual function mediates the association between dementia and functional activities of daily living.

**Methods:**

A retrospective, cross‐sectional design that analyzed the mediating effects of visual functions on the association between dementia and functional activity of daily living. We retrieved 2,523 adults from the 3,817 survey participants in the 2022 National Health and Aging Trends Study. We utilized a series of path analysis models and estimated the mediating effects of visual functions between dementia and functional daily activities. We used the older adults with and without dementia as the independent variables. The mediators were total scores of three visual function variables (distance acuity, near acuity, contrast sensitivity). The dependent variables were the total scores of ADLs (e.g., dressing, toileting) and IADLs (e.g., laundry, shopping).

**Results:**

Among the 2,523 older adults, 201 (8.0%) older adults with dementia, and 1,446 (57.3%) females. In the association between dementia and ADLs, the distance acuity revealed a significant mediation effect of 9.9% (direct effect β = 0.1875, p <0.0001, indirect effect β = 0.0205, p <0.0001), and IADLs showed a significant mediating effect of 8.8% (direct effect β = 0.2095, p <0.0001, indirect effect β = 0.0.0201, p <0.0001). In the association between dementia and ADLs, the contrast sensitivity revealed a significant mediation effect of 11.2% (direct effect β = 0.1847, p <0.0001, indirect effect β = 0.0233, p <0.0001), and IADLs showed a significant mediating effect of 11.6% (direct effect β = 0.2030, p <0.0001, indirect effect β = 0.0266, p <0.0001).

**Conclusions:**

The study findings revealed the importance of visual function between dementia, ADL, and IADLs. Our findings indicated that healthcare professionals would need to provide interventions that consider visual functions to maintain functional activities of daily living in older adults with dementia.